# Supplementary material for: Unveiling the Bioactive Potential of the Invasive Jellyfish Phyllorhiza punctata Through Integrative Transcriptomic and Proteomic Analyses
Source: Biomolecules. 2025 Aug 4;15(8):1121. doi: 10.3390/biom15081121 (PMC12383608; doi:10.3390/biom15081121)
Supplement: Supplementary file 1 [file biomolecules-15-01121-s001.zip › Suplementary Material/FileS3/PEPFOLD4/XP_065062238_5241/clusters.html]

 PEP-FOLD clusters


Download archive of all models  here   

|  |  |  |
| --- | --- | --- |
| # | file | sOPEP |
| # cluster 1 - Size: 14 |  |  |
|  | XP\_065062238\_5241-model1 | -23.4633 |
|  | XP\_065062238\_5241-model1.1 | -23.3975 |
|  | XP\_065062238\_5241-model1.2 | -23.3072 |
|  | XP\_065062238\_5241-model1.3 | -23.2661 |
|  | XP\_065062238\_5241-model1.4 | -22.9965 |
|  | XP\_065062238\_5241-model1.5 | -22.9558 |
|  | XP\_065062238\_5241-model1.6 | -22.8332 |
|  | XP\_065062238\_5241-model1.7 | -22.7462 |
|  | XP\_065062238\_5241-model1.8 | -22.6761 |
|  | XP\_065062238\_5241-model1.9 | -22.661 |
|  | XP\_065062238\_5241-model1.10 | -22.6382 |
|  | XP\_065062238\_5241-model1.11 | -22.6364 |
|  | XP\_065062238\_5241-model1.12 | -22.5859 |
|  | XP\_065062238\_5241-model1.13 | -22.4272 |
| # cluster 2 - Size: 86 |  |  |
|  | XP\_065062238\_5241-model2 | -22.9156 |
|  | XP\_065062238\_5241-model2.1 | -22.912 |
|  | XP\_065062238\_5241-model2.2 | -22.7963 |
|  | XP\_065062238\_5241-model2.3 | -22.7896 |
|  | XP\_065062238\_5241-model2.4 | -22.7896 |
|  | XP\_065062238\_5241-model2.5 | -22.7896 |
|  | XP\_065062238\_5241-model2.6 | -22.7846 |
|  | XP\_065062238\_5241-model2.7 | -22.7846 |
|  | XP\_065062238\_5241-model2.8 | -22.7846 |
|  | XP\_065062238\_5241-model2.9 | -22.7846 |
|  | XP\_065062238\_5241-model2.10 | -22.7846 |
|  | XP\_065062238\_5241-model2.11 | -22.7711 |
|  | XP\_065062238\_5241-model2.12 | -22.7711 |
|  | XP\_065062238\_5241-model2.13 | -22.7451 |
|  | XP\_065062238\_5241-model2.14 | -22.7451 |
|  | XP\_065062238\_5241-model2.15 | -22.7451 |
|  | XP\_065062238\_5241-model2.16 | -22.7451 |
|  | XP\_065062238\_5241-model2.17 | -22.7451 |
|  | XP\_065062238\_5241-model2.18 | -22.7451 |
|  | XP\_065062238\_5241-model2.19 | -22.7151 |
|  | XP\_065062238\_5241-model2.20 | -22.7018 |
|  | XP\_065062238\_5241-model2.21 | -22.6857 |
|  | XP\_065062238\_5241-model2.22 | -22.6857 |
|  | XP\_065062238\_5241-model2.23 | -22.6857 |
|  | XP\_065062238\_5241-model2.24 | -22.6857 |
|  | XP\_065062238\_5241-model2.25 | -22.6857 |
|  | XP\_065062238\_5241-model2.26 | -22.6857 |
|  | XP\_065062238\_5241-model2.27 | -22.6722 |
|  | XP\_065062238\_5241-model2.28 | -22.6722 |
|  | XP\_065062238\_5241-model2.29 | -22.6623 |
|  | XP\_065062238\_5241-model2.30 | -22.6622 |
|  | XP\_065062238\_5241-model2.31 | -22.6575 |
|  | XP\_065062238\_5241-model2.32 | -22.6572 |
|  | XP\_065062238\_5241-model2.33 | -22.6572 |
|  | XP\_065062238\_5241-model2.34 | -22.6561 |
|  | XP\_065062238\_5241-model2.35 | -22.649 |
|  | XP\_065062238\_5241-model2.36 | -22.6486 |
|  | XP\_065062238\_5241-model2.37 | -22.6357 |
|  | XP\_065062238\_5241-model2.38 | -22.6328 |
|  | XP\_065062238\_5241-model2.39 | -22.6242 |
|  | XP\_065062238\_5241-model2.40 | -22.622 |
|  | XP\_065062238\_5241-model2.41 | -22.622 |
|  | XP\_065062238\_5241-model2.42 | -22.6147 |
|  | XP\_065062238\_5241-model2.43 | -22.6147 |
|  | XP\_065062238\_5241-model2.44 | -22.6092 |
|  | XP\_065062238\_5241-model2.45 | -22.6092 |
|  | XP\_065062238\_5241-model2.46 | -22.6079 |
|  | XP\_065062238\_5241-model2.47 | -22.5961 |
|  | XP\_065062238\_5241-model2.48 | -22.5961 |
|  | XP\_065062238\_5241-model2.49 | -22.5961 |
|  | XP\_065062238\_5241-model2.50 | -22.5961 |
|  | XP\_065062238\_5241-model2.51 | -22.5925 |
|  | XP\_065062238\_5241-model2.52 | -22.5907 |
|  | XP\_065062238\_5241-model2.53 | -22.5811 |
|  | XP\_065062238\_5241-model2.54 | -22.5811 |
|  | XP\_065062238\_5241-model2.55 | -22.5692 |
|  | XP\_065062238\_5241-model2.56 | -22.5692 |
|  | XP\_065062238\_5241-model2.57 | -22.5606 |
|  | XP\_065062238\_5241-model2.58 | -22.5606 |
|  | XP\_065062238\_5241-model2.59 | -22.5606 |
|  | XP\_065062238\_5241-model2.60 | -22.5606 |
|  | XP\_065062238\_5241-model2.61 | -22.5606 |
|  | XP\_065062238\_5241-model2.62 | -22.5606 |
|  | XP\_065062238\_5241-model2.63 | -22.5606 |
|  | XP\_065062238\_5241-model2.64 | -22.5603 |
|  | XP\_065062238\_5241-model2.65 | -22.5576 |
|  | XP\_065062238\_5241-model2.66 | -22.5444 |
|  | XP\_065062238\_5241-model2.67 | -22.5422 |
|  | XP\_065062238\_5241-model2.68 | -22.5323 |
|  | XP\_065062238\_5241-model2.69 | -22.5216 |
|  | XP\_065062238\_5241-model2.70 | -22.5153 |
|  | XP\_065062238\_5241-model2.71 | -22.5139 |
|  | XP\_065062238\_5241-model2.72 | -22.5076 |
|  | XP\_065062238\_5241-model2.73 | -22.5062 |
|  | XP\_065062238\_5241-model2.74 | -22.4959 |
|  | XP\_065062238\_5241-model2.75 | -22.4671 |
|  | XP\_065062238\_5241-model2.76 | -22.4633 |
|  | XP\_065062238\_5241-model2.77 | -22.4612 |
|  | XP\_065062238\_5241-model2.78 | -22.435 |
|  | XP\_065062238\_5241-model2.79 | -22.4254 |
|  | XP\_065062238\_5241-model2.80 | -22.3387 |
|  | XP\_065062238\_5241-model2.81 | -22.3387 |
|  | XP\_065062238\_5241-model2.82 | -22.3387 |
|  | XP\_065062238\_5241-model2.83 | -22.3108 |
|  | XP\_065062238\_5241-model2.84 | -22.2878 |
|  | XP\_065062238\_5241-model2.85 | -22.2631 |
|  |  |  |
